# Fast and exact quantification of motif occurrences in biological sequences

**DOI:** 10.1186/s12859-021-04355-6

**Published:** 2021-09-18

**Authors:** Mattia Prosperi, Simone Marini, Christina Boucher

**Affiliations:** 1 Data Intelligence Systems Lab, Department of Epidemiology, College of Public Health and Health Professions and College of Medicine, University of Florida, Gainesville, FL USA; 2 Department of Computer and Information Science and Engineering, University of Florida, Gainesville, FL USA

**Keywords:** Bioinformatics, Motifs, Probability distribution, Markov model

## Abstract

**Background:**

Identification of motifs and quantification of their occurrences are important for the study of genetic diseases, gene evolution, transcription sites, and other biological mechanisms. Exact formulae for estimating count distributions of motifs under Markovian assumptions have high computational complexity and are impractical to be used on large motif sets. Approximated formulae, e.g. based on compound Poisson, are faster, but reliable *p* value calculation remains challenging. Here, we introduce ‘motif_prob’, a fast implementation of an exact formula for motif count distribution through progressive approximation with arbitrary precision. Our implementation speeds up the exact calculation, usually impractical, making it feasible and posit to substitute currently employed heuristics.

**Results:**

We implement motif_prob in both Perl and C+ + languages, using an efficient error-bound iterative process for the exact formula, providing comparison with state-of-the-art tools (e.g. MoSDi) in terms of precision, run time benchmarks, along with a real-world use case on bacterial motif characterization. Our software is able to process a million of motifs (13–31 bases) over genome lengths of 5 million bases within the minute on a regular laptop, and the run times for both the Perl and C+ + code are several orders of magnitude smaller (50–1000× faster) than MoSDi, even when using their fast compound Poisson approximation (60–120× faster). In the real-world use cases, we first show the consistency of motif_prob with MoSDi, and then how the p-value quantification is crucial for enrichment quantification when bacteria have different GC content, using motifs found in antimicrobial resistance genes. The software and the code sources are available under the MIT license at https://github.com/DataIntellSystLab/motif_prob.

**Conclusions:**

The motif_prob software is a multi-platform and efficient open source solution for calculating exact frequency distributions of motifs. It can be integrated with motif discovery/characterization tools for quantifying enrichment and deviation from expected frequency ranges with exact *p* values, without loss in data processing efficiency.

## Background

Motif discovery and characterization are important for the study of gene evolution, duplication, transcription sites, and protein identification [1], as well as of genetic diseases caused by unstable repeat expansion [2, 3].

Several tools have been developed for de novo motif discovery [4–6]—including discriminative regular expression motif elicitation (DREME), hypergeometric optimization of motif enrichment (HOMER), multiple expectation maximizations for motif elicitation (MEME), the memetic framework for motif discovery (MFMD), peak-motifs, prosampler, regulatory sequence analysis tools (RSAT), Trawler Web, and Weeder—either generic or specialized, e.g. for ChIP-seq data [7–15].

Assessing the statistical significance of motif enrichment is a fundamental and challenging step of motif discovery, and can severely hamper downstream analytics. Kiesel et al. [16] pointed out that *p* values *“Small enrichment factors can occur frequently in practice simply due to an imperfect background model that slightly underestimates the expected frequency of occurrence”*. In addition, *p* values are crucial not only in the discovery phases, but also in motif comparison and motif-motif similarity studies [17]. The classical definition of the motif enrichment problem (in terms of differences among motifs occurrences within background genome contents) has been proven to be NP-hard [18]. The *p* value calculation is not straightforward, and requires making assumptions on a background model of base frequencies and co-occurrence in order to derive a distribution of motif occurrences in reference genomes [19]. Several formulae—approximated and exact—and algorithms for estimating motif count distributions have been devised and implemented [20–28]. Exact formulae for estimating count distributions of motifs under Markovian assumptions have high computational complexity and are impractical to be used on large data sets. Approximated formulae, e.g. based on compound Poisson, are faster, but reliable *p* value calculation remains challenging [19, 25]. Thus, methods for *p* value estimation can be a bottleneck in large-scale projects. HOMER, Weeder and Peak-motifs do not report motif statistical significance, MEME uses an approximation approach (very conservative), later improved by DREME and the new simple, thorough, rapid, enriched motif elicitation (STREME) [10, 15], and MFMD uses information content score and complexity scores [29].

A software that provides a comprehensive occurrence and probability estimation is the bioinformatics toolkit for Motif Statistics and Discovery (MoSDi) by Marschall [30], written in Java, featuring models based on the approximated compound Poisson and *n*th level Markov order, as well as (quasi-)exact combinatorial formulae to reduce computational complexity (https://bitbucket.org/tobiasmarschall/mosdi). Another tool is motifcounter [31], an R-Bioconductor library implementing existing methods [27, 32], as well as an improvement on the compound Poisson model. One limitation of these programs is that calculation of occurrence distribution—even using the fast compound Poisson—becomes impractical with longer motifs (10+) and longer reference genomes (millions of bases), besides large motif datasets.

Prosperi et al. [28] provided an exact formula for counting the distribution of strings that do not overlap with themselves (i.e. *non-clumpable*), coupled with a mathematical demonstration of its validity, under both Bernoullian and Markovian assumptions. The calculation of the formula was exponential in the genome length by the length of the motif, but the authors demonstrated that it could be calculated efficiently within an arbitrary tolerance level.

This software article describes “motif_prob”, a count distribution tool suitable for long motifs and long reference genomes, implementing the exact method by Prosperi et al. [28] with the efficient error-bound algorithm. In addition to the relevance of this software piece for large-scale processing, another motivation for our work is that the majority of probability distribution or *p* value calculators, even the most recent ones, use heuristics. To our knowledge, the formula by Prosperi et al. is still among the most efficient for *exact* calculation. The proposed motif_prob implementation thus makes exact quantification suitable with large scale projects, and posits to substitute currently employed heuristics. We compare motif_prob with other tools in terms of run time and precision, showing that its exact algorithm is several orders of magnitude faster even than the approximated methods, and finally we describe use cases for long motifs in bacteria.

## Methods

### Theoretical formulation

The exact formula by Prosperi et al. [28], for the calculation of the frequency distribution *j* of a string of length *m* within a text of length *n* (*m* < *n*) over alphabet *k*, under the Markovian model, is

1 $$\begin{array}{*{20}c} {P\left( {j,m,n} \right) = P\left( S \right)^{j} \mathop \sum \limits_{z = 1}^{{\left| {C_{n,m,j} } \right|}} \mathop \prod \limits_{y = 1}^{j + 1} P\left( {S_{{0,d_{yz} }} } \right),} \\ \end{array}$$

where *P*(*S*) = *P*(*a*_*1*_) · *P*(*a*_*2*_ | *a*_*1*_) · … · *P*(*a*_*m*−*1*_ | *a*_*m*_), *P*(*S*_*0,n*_) = *P*(*S*_*0,n*−*1*_) − *P*(*S*) · *P*(*S*_*0,n*−*m*_), *S*_*0,n*_ = *S*_*0,n*−*1*_* · k* − *P(S) · k*^*m*^* · S*_*0,n*−*m*_, *d*_*1*_* … d*_*j*+*1*_ are the lengths of the *j* + *1* segments where the *j* strings divide the text of length *n* in exact configurations with *d*_*1*_ + *···* + *d*_*j*+*1*_ = *n − mj*, and

2 $$\begin{array}{*{20}c} {\left| {C_{n,m,j} } \right| = \left( {\begin{array}{*{20}c} {n + j\left( {1 - m} \right)} \\ {n - mj} \\ \end{array} } \right).} \\ \end{array}$$

Formula (1) has a complexity of O(*n*^*j*^), which becomes quickly intractable. However, by defining *R* = *P*(*S*_0,*n*+1_)/*P*(*S*_0,*n*_) as a constant, Prosperi et al*.* show that for any positive (arbitrarily small) number *ε*, there is an index *η*_*ε*_ such that for every *η* > *η*_*ε*_ then

3 $$\begin{array}{*{20}c} {P\left( {S_{0,n + x} } \right) \sim P\left( {S_{0,n} } \right) \cdot R^{x} . } \\ \end{array}$$

By using this approximation, the summation of the original formula can be reduced to a single step, and calculations can be stopped when the ratio *P*(*S*_0,*n*_)/*P*(*S*_0,*n*−1_) reaches a desired level of tolerance *ε*. Specifically, after plugging the iterative approximation (3) in (1), we obtain the final formula

4 $$\begin{array}{*{20}c} {P\left( {j,m,n} \right) \sim P\left( S \right)^{j} \cdot R^{{n - mj - n_{\varepsilon } \left( {j + 1} \right)}} \cdot P\left( {S_{{\left( {0,n_{\varepsilon } } \right)}} } \right)^{j + 1} \cdot \left( {\begin{array}{*{20}c} {n + j\left( {1 - m} \right)} \\ {n - mj} \\ \end{array} } \right).} \\ \end{array}$$

We note that *P*(*j, m, n*) is the same irrespective of the position of the nucleotides in a query string, e.g. AACCC and CCCAA have the same probability. This property permits to extrapolate a probability for clumpable strings by permutation, e.g. ACCA into CCAA, although the value is not guaranteed given possible overlap. Another way is to replace the first or the last character with another one that has the same frequency. All details on the derivation of the exact formula and the proof for its progressive approximation, along with comparison against other state-of-art algorithms, can be found in the original work by Prosperi et al. [28].

### Implementation

Two different implementations are produced: one in Perl and another in C++. Both programs take the same input and parameters, namely: (1) a query string or multiple strings to be analyzed; (2) the length of the reference genome; and (3) the nucleotide frequencies of the genome. In alternative to the genome length and nucleotide frequencies, a FASTA file containing the genome string can be passed as input to the program. The output file reports—for each motif—the count distribution and other summary information including a flag for clumped strings, string probability, and statistics on the precision and tolerance levels.

Since the computational complexity of the formula is exponential, motif occurrences are calculated at increasing counts until the occurrence probability becomes lower than given a tolerance level *ε*, or the upper limit of counts *j* is reached. We also control estimates at each iteration in order to avoid issues with floating point operations when frequency/length ratios diverge, and to handle relatively ill-posed configurations. Given the motif *m* and genome *g* lengths, one can set a tolerance level *ε* such that *P*(0, *m, n*) > (1 − *ε*), and in general each case where (1 − *P*(*S*))^(m−m+1)^ > (1 − *ε*). This is equal to (*n* − *m* + 1)∙log(1 − *P*(*S*)) > log(1 − *ε*), which implies *n* > *m* − 1 + log(1 − *ε*)/log(1 − *P*(*S*)). In the source code, we have set *ε* to 10^−7^ and *j* to 500. Further, we implement the calculation of the expected number of strings and the motif’s (stationary) occurrence probability at any text position, according to Robin et al. [33].

**Fig. 1:**
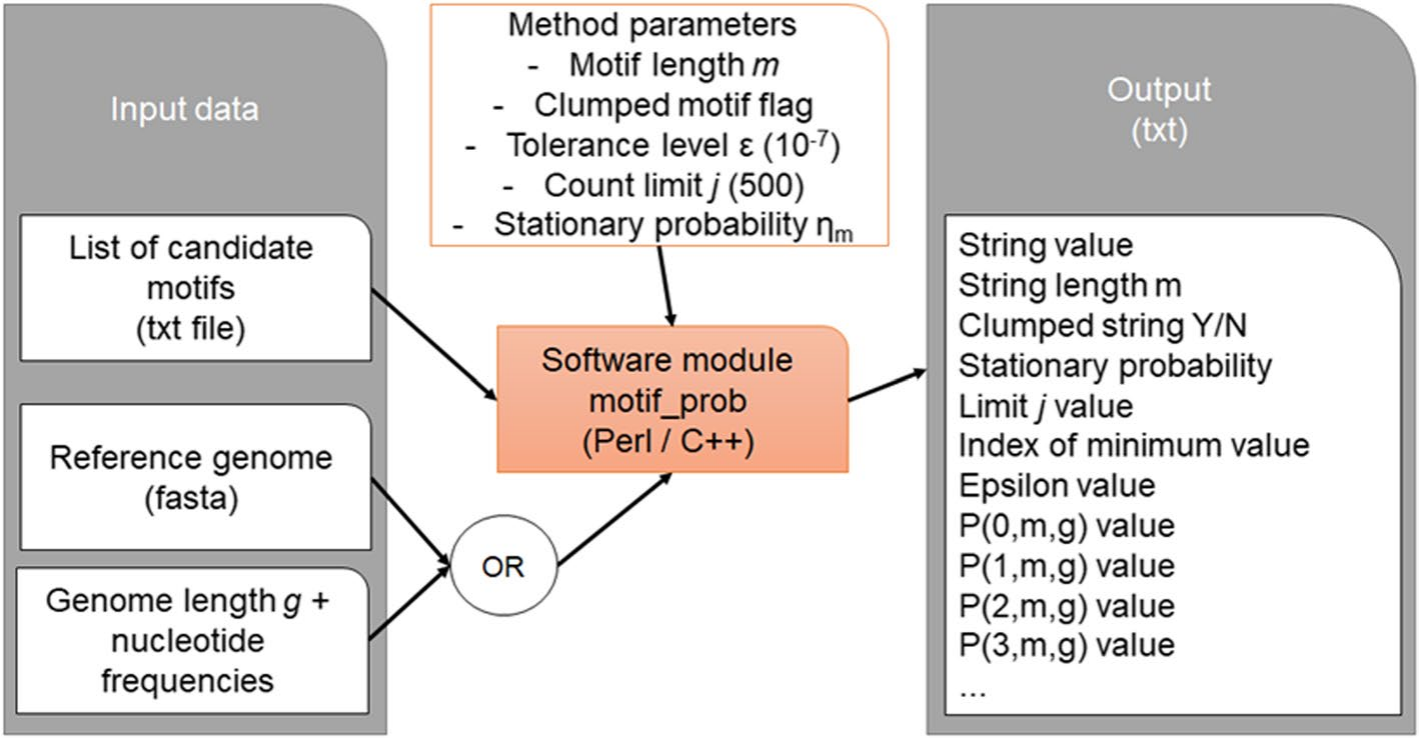
Flowchart of the data processing pipeline for motif_prob, with input/output specifications and program parameters

Figure 1 provides a flowchart of the data processing pipeline, showing the required input specifications, the method’s internal parameters, and the output fields.

The source code, documentation, sample datasets, and executable files are available under the MIT license at https://github.com/DataIntellSystLab/motif_prob.

## Results

**Fig. 2:**
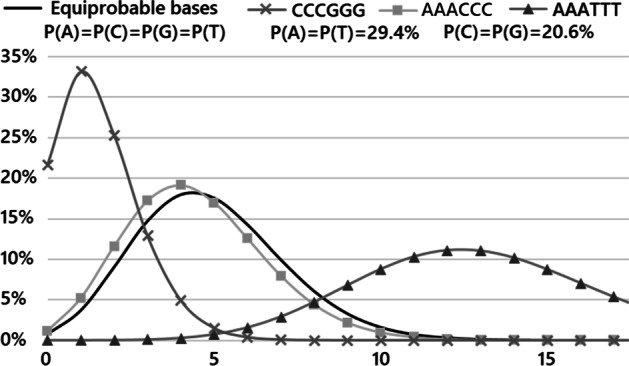
Application output for motif sequences of length 6 over a genome of length 20,000, and different nucleotide frequencies

An example of the occurrence distribution for motif query sequences of length 6, calculated on a randomly generated genome of 20,000 bases, varying the nucleotide frequencies, is illustrated in Fig. 2. The difference between the equiprobable base and the more general case is evident and demonstrates how the background distribution affects the *p* value calculation (see real-world use case after the benchmarks).

Table 1 shows run time benchmarks on different motif length and motif set size configurations, executed on a laptop machine with Intel(R) Core(TM) i9-10885H CPU @ 2.4 GHz, 32 GB RAM. Both the Perl and the C++ programs exhibit run times several orders of magnitude smaller than MoSDi, even when the latter is executed with the fast compound Poisson approximation. We set a maximum processing time of 30 min for datasets up to 400,000 motifs, and MoSDi can process them only with smaller values of *k* and the approximated model, while the exact model is not feasible for most of datasets. The C++ implementation is the fastest, and the expected run time increase due to higher motif lengths is well compensated by the implementation setup.

**Table 1:** Run time (mm:ss) of the Perl and C++ programs compared to MoSDi (exact and approximated using compound Poisson) for calculating the occurrence distribution for *s* motif query sequences of length *m* (13–31) over a reference genome of 5 million bases

No. of motifs *s*	Motif length *m*	C++	Perl	MoSDi exact (10/500)	MoSDi approx (10/500)
10	13	00:00	00:00	00:47/17:50	00:00/00:00
20,000	13	00:01	00:07	xx:xx/xx:xx	01:16/02:24
50,000	13	00:03	00:18	xx:xx/xx:xx	03:14/06:24
200,000	13	00:15	01:12	xx:xx/xx:xx	13:34/27:40
400,000	13	00:35	02:20	xx:xx/xx:xx	28:05/xx:xx
1,000,000	13	00:85	05:45	xx:xx/xx:xx	xx:xx/xx:xx
10	31	00:00	00:00	01:47/xx:xx	00:00/00:01
20,000	31	00:02	00:07	xx:xx/xx:xx	15:34/16:41
50,000	31	00:04	00:17	xx:xx/xx:xx	xx:xx/xx:xx
200,000	31	00:16	01:09	xx:xx/xx:xx	xx:xx/xx:xx
400,000	31	00:29	02:26	xx:xx/xx:xx	xx:xx/xx:xx
1,000,000	31	00:32	05:18	xx:xx/xx:xx	xx:xx/xx:xx

Runs lasting over 30:00 were stopped

In terms of precision, we compare the exact probability values yielded by our program with both the compound Poisson and the exact estimates of MoSDi (allowing it to switch automatically to standard/doubling algorithms to improve run time). As previously described, usually the largest errors appears near the probability mass points [28]. For all motif lengths combinations of 4 bases, over a 10,000 bases reference genome, on average the peak probability values of MoSDi and motif_prob exact differ by two orders of magnitude, e.g. if the peak probability is in the range of 10^−2^ then the observed absolute difference is 10^−4^. The difference with the compound Poisson approximation is larger, on average double than the exact, but the relative ratio it is still one-two orders of magnitude smaller than the actual values. The difference becomes smaller as the sequence lengths increase.

**Fig. 3:**
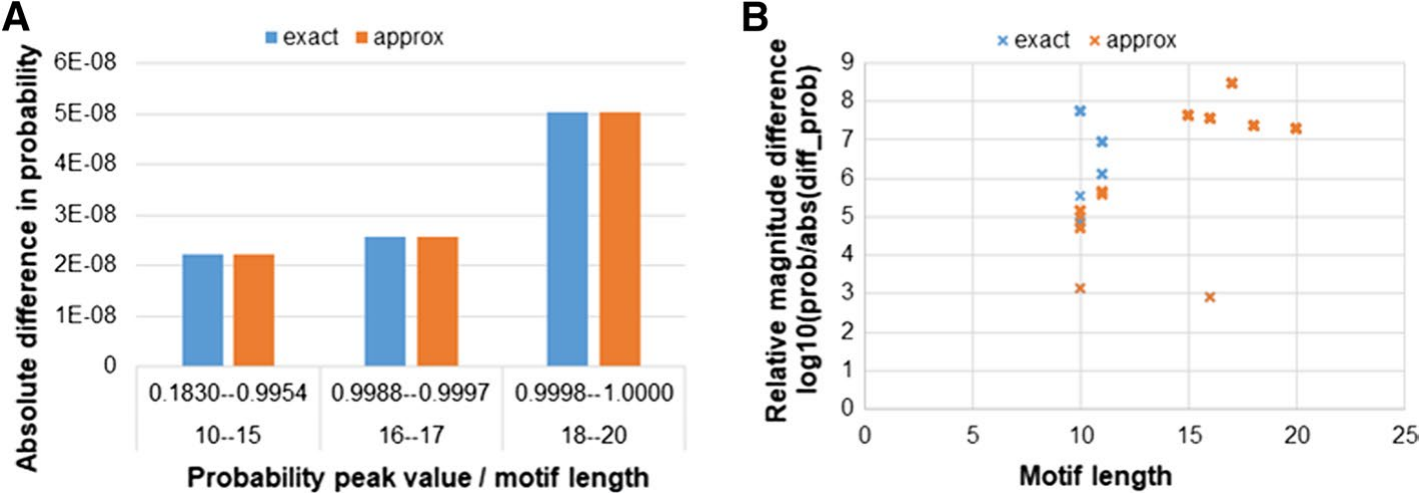
Comparison between motif_prob and MoSDi (exact and approximated with compound Poisson) in terms of concordance of probability estimates. Panel **A** shows the absolute difference in peak probability values, stratified by motif length and probability mass value, while panel **B** shows the relative magnitude difference by motif length

We further test the concordance among MoSDi and motif_prob using a real motif dataset, the library of DNA-binding site matrices for *Escherichia coli* (https://arep.med.harvard.edu/ecoli_matrices/), which contains 802 motifs from 67 housekeeping genes for a median motif length of 26 (interquartile range, IQR 20–29). We consider motifs length within 20 bases to be able to estimate non-near-zero probabilities on the genome length of *Escherichia coli*. The final set includes 230 motifs with a median length 16 (IQR 15–18). The median (IQR) difference between MoSDi and motif_prob exact overall is 2.6·10^−8^ (2.2·10^−8^–5.0·10^−8^), while for all probabilities where the center of mass is not zero (median 0.18), it is 3.8·10^–8^ (3.3·10^−9^–2.6·10^−7^). Once again, the differences with the approximated estimation are larger but of the same level of magnitude. Figure 3 illustrates the absolute difference in probability between motif_prob and MoSDi (exact/compound Poisson) as well as the relative magnitude difference, expressed as the log10(Prob_motif_prob_/abs(Prob_motif_prob_ − Prob_MoSDi_)), which well highlights how the difference between the two exact methods (and the compound Poisson too, although larger) is negligible with respect to the actual probability estimates.

As a final use case, we investigate the distribution of frequencies of antimicrobial resistance gene signatures found in bacteria under different GC content. Drug resistance mechanisms in bacteria involve acquisition of genes, often via mobile genetic elements, and in some cases changes within core housekeeping genes. A number of algorithms use *k*-mers, i.e. motifs of fixed *k* length, to classify antimicrobial resistance [34], as they can be handled efficiently through ad hoc data structures suitable to process high-throughput data. But assessing the importance of a *k*-mer with respect to their frequency in drug resistance genes is not straightforward; one issue is that bacteria and genes can have very different GC content [35]. When the GC content varies, the probability distributions of motif occurrence can change over a broad range (given also the underlying, individual A, C, G, and T content), and thus the *p* values of over- or under-representation. To show how the quantification can have large variance, we analyze *k*-mers from antimicrobial resistance genes collected in the MEGARes 2.0 database [36]. MEGARes contains 7868 genes, with an average gene length of 1030.29 nucleotide bases, 57 different antibiotic resistance classes, and 220 distinct resistance mechanisms.

From MEGARes, we select all the 3911 genes conferring resistance to beta-lactamase; we then identify all 13-mers, for a total of 453,308 motifs (50% GC content). In Table 2, we show how the count probability distribution of the 13-mers in MEGARes’ beta-lactamase genes changes among bacterial species present in the human microbiome of respiratory tract [37], where we select uniformly 18 species on the basis of their GC content. The median probability of finding the aforementioned 13-mers at least once varies between 93 and 99%, and even species with a similar GC content can show different medians and interquartile ranges, such as *Stomatobaculum longum* (55% GC content, median *p* = 97%) and *Kluyvera intermedia* (52% GC content, median *p* = 93%). This variability is due to: the individual nucleotide content, which can differ even when the GC content is the same, and it directly affects the distribution (see also Fig. 2); the genome length; and the nucleotide content of the query motifs.

**Table 2:** Median (interquartile range, IQR) probability of finding at least once 13-mer motifs (top-frequent among beta-lactamase resistance genes) in the MEGARes database over different bacterial species characterized by heterogeneous GC content

Species	Genome length	GC content	Median (IQR) probability
*Nocardioides Salarius*	4,429,322	0.73	0.98 (0.94–1)
*Enhydrobacter aerosaccus*	6,767,089	0.65	0.93 (0.87–0.98)
*Paraburkholderia ginsengisol*	6,541,884	0.64	0.93 (0.87–0.97)
*Neisseria shayeganii*	2,419,744	0.58	0.97 (0.95–0.98)
*Stomatobaculum longum*	2,308,581	0.55	0.97 (0.96–0.98)
*Kluyvera intermedia*	4,938,529	0.52	0.93 (0.92–0.94)
*Buttiauxella noackiae*	4,766,673	0.49	0.93 (0.93–0.93)
*Megasphaera micronuciformis*	1,765,374	0.45	0.97 (0.97–0.98)
*Oribacterium sinus*	2,727,518	0.43	0.96 (0.95–0.98)
*Prevotella jejuni*	3,913,006	0.42	0.95 (0.92–0.97)
*Prevotella melaninogenica*	3,168,282	0.4	0.96 (0.94–0.98)
*Streptococcus pseudopneumoniae*	2,195,458	0.4	0.97 (0.95–0.99)
*Veillonella rogosae*	2,187,106	0.39	0.97 (0.95–0.99)
*Lachnoanaerobaculum orale*	2,799,073	0.38	0.97 (0.94–0.99)
*Catonella morbi*	3,477,404	0.38	0.96 (0.93–0.99)
*Staphylococcus argenteus*	2,753,898	0.32	0.98 (0.95–0.99)
*Leptotrichia wadei*	2,337,418	0.29	0.98 (0.96–1)
*Fusobacterium nucleatum*	2,455,060	0.26	0.99 (0.97–1)

## Conclusion

The motif_prob software is a multi-platform, open source, efficient solution for calculating exact frequency distributions of (long) motif occurrences in reference genomes using high-throughput data. We showed how our code estimates are consistent with other, slower, exact calculations, and how the run times of our code (both Perl and C++) are competitive even with the non-exact compound Poisson approximation. Specifically, motif_prob is 50–1000× faster than MoSDi exact and 60–120× faster than MoSDi compound Poisson.

The current implementation is limited to non-clumpable strings, although it extrapolates a probability for clumpable strings by permutation. As future development of our work we foresee to develop an exact formula for clumpable strings and to extend the approach to generalize over motifs that can include nucleotide changes, insertions or deletions.

In conclusion, our tool can be effectively used in conjunction with motif discovery suites that process high-throughput data, allowing them to compute exact count distributions and associated *p* values without loss of run time performance, instead of relying on to approximations.

## Availability and requirements

*Project name*: motif_prob

*Project home page*: https://github.com/DataIntellSystLab/motif_prob

*Operating system(s)*: Multi-platform (UNIX/Linux/Mac, Windows)

*Programming language*: Perl, C++

*Other requirements*: None

*License*: MIT

*Any restrictions to use by non-academics:* Permissible under the terms of the MIT license.

## Data Availability

The datasets generated and/or analysed during the current study are available in the motif_prob GitHub repository (https://github.com/DataIntellSystLab/motif_prob), the MEGARes database (https://megares.meglab.org/) and the website for DNA-binding site matrices for Escherichia coli (https://arep.med.harvard.edu/ecoli_matrices/).

## Abbreviations

DREME: Discriminative regular expression motif elicitation
HOMER: Hypergeometric optimization of motif enrichment
MEME: Multiple expectation maximizations for motif elicitation
MFMD: Memetic framework for motif discovery
RSAT: Regulatory sequence analysis tools
STREME: Simple, thorough, rapid, enriched motif elicitation
MoSDi: Motif statistics and discovery
mm:ss: Minutes:seconds
IQR: Interquartile range
